# Understanding structure/function relationships in nitrifying microbial communities after cross-transfer between freshwater and seawater

**DOI:** 10.1038/s41598-021-82272-7

**Published:** 2021-02-03

**Authors:** Blanca M. Gonzalez-Silva, Kjell Rune Jonassen, Ingrid Bakke, Kjetill Østgaard, Olav Vadstein

**Affiliations:** 1 Department of Biotechnology and Food Science, Faculty of Natural Sciences and Technology, NTNU-Norwegian University of Science and Technology, Sem Saelands v. 6/8, N-7491 Trondheim, Norway; 2 Present Address: Department of Civil and Environmental Engineering, NTNU-Norwegian University of Science and Technology, S. P. Andersens veg 5, N-7031 Trondheim, Norway; 3 Present Address: VEAS, Bjerkåsholmen 125, 3470 Slemmestad, Oslo, Norway

**Keywords:** Biotechnology, Molecular biology, Environmental sciences

## Abstract

In this study, nitrification before and after abrupt cross-transfer in salinity was investigated in two moving bed biofilm reactors inoculated with nitrifying cultures that had adaptation to freshwater (FR) and seawater salinities (SR). FR and SR MBRRs were exposed to short and long term cross-transfer in salinity, and the functional capacity of nitrifying microbial communities was quantified by the estimation of ammonia and nitrite oxidation rates. Salinity induced successions were evaluated before and after salinity change by deep sequencing of 16S rRNA gene amplicons and statistical analysis. The bacterial community structure was characterized and Venn diagrams were included. The results indicated that after salinity cross-transfer, the FR was not significantly recovered at seawater salinity whereas SR showed high resistance to stress caused by low-salt. Succession and physiological plasticity were the main mechanisms of the long-term adaption of the nitrifying communities exposed to abrupt salinity changes. Independently of salinity, some nitrifiers presented high physiological plasticity towards salinity and were very successful at both zero and full seawater salinity. SR culture is robust and suitable inoculum for ammonium removal from recirculating aquaculture systems and industrial wastewaters with variable and fast salinity changes. Our findings contradict the current perspective of the significance of salinity on the structure of nitrifying communities.

## Introduction

Nitrification, the sequential aerobic oxidation of ammonia to nitrate via nitrite, is a central nitrogen (N) cycling process^1^ and is particularly susceptible to inhibition by salt^2^. Therefore, one important challenging aspect of wastewater treatment is the management of salinity effluents^3^. Origins of such wastewater streams are e.g. fish canning industries^4,5^, seafood processing^6^, and marine fish or shrimp aquaculture^7^. A global trend in the land-based aquaculture industry is the change from flow-through to recirculating aquaculture systems (RAS). During the production of Atlantic salmon (*Salmo salar*), freshwater is used during the young life stages (from hatching to smolt), and seawater is normally used during later life stages (post-smolt)—also for land-based production in RAS^8,9^. As brackish and saltwater RAS develop very rapidly, there is an urgent need for more research to improve our understanding and to optimizing biofiltration at different salinities^10^ and after abrupt salinity changes.

Variable salinity leads to osmotic stress, and the consequence can be loss of metabolic enzyme activity and inhibition of the growth of microorganisms^11^. To reduce the salt stress on bacterial metabolism during shift-up, different acclimatization strategies of freshwater nitrifiers to high salinity^9,12^ as well as utilization of halophilic nitrifying microbial consortia^7^ have been reported. However, studies on the structure and function relationships of nitrifying biofilms exposed to fast (shift-up or down) salinity change in long-term are scarce in the literature. Need for a fundamental understanding of the recovery time and community dynamics of nitrifying bacteria exposed to drastic salinity changes is important in order to predict future changes in the community structure, nitrifying activity, and removal performance. Of special interest is whether long term adaptation is due to physiological adaptation or a succession towards species that are more adapted to the new salinity.

In a previous study, we reported a comprehensive insight on the composition of the ammonium oxidizing bacteria (AOB) and nitrite-oxidizing bacteria (NOB) in the nitrifying microbial community of the three moving bed biofilm reactors (MBBRs), inoculated with cultures that had a long term adaptation to freshwater (FR), brackish water (BR), and seawater (SR) salinities^13^. Furthermore, a sophisticated and expanded Venn diagram was introduced as a useful tool to visualize the distribution of shared AOB and NOB OTUs between the three MBRRs at different salinity. The present study expands that work to explore the effect of an abrupt change in salinity of the two different nitrifying microbial communities, FR and SR, that were long term adapted (years) to their native salinity. Therefore FR and SR cultures used in our previous study were exposed to long term cross-transfer in salinity, and their robustness against salt stress was quantified. The functional capacity of these two nitrifying microbial communities was followed by the estimation of ammonia and nitrite oxidation rates, the structure of the microbial community and Venn diagrams to reveal common and chaired species inventory and their relative abundance. The salinity-induced successions were evaluated before and after salinity change by 16S rRNA gene amplicons and statistical analysis. The significance of salinity on the structure of nitrifying communities was investigated, and our finding contradicts the current perspective of salinity as a strong selective force.

## Materials and methods

### Media composition

A freshwater and a seawater growth media were prepared with tap water and seawater (with 33‰, from Trondheimsfjord), respectively, and macro and micronutrients with the following composition (g L^−1^): (NH_4_)_2_ SO_4_, varying; K_2_HPO_4_ (Freshwater reactor; FR reactor), 0.4; NaH_2_PO_4_·7H_2_O (Seawater reactor; SR reactor), 0.05 (S); NaHCO_3_, 1; 10 mL L^−1^ trace solution containing (g L^−1^): MgSO_4_·7H_2_O, 2.5; MnCl_2_·4H_2_O, 0.55; CaCl_2_·2H_2_O 0.05; ZnCl_2_, 0.068; CoCl_2_·6H_2_O 0.12; NiCl_2_·6H_2_O 0.12; FeCl_2_, 0.4. Prior to use, the seawater medium was filtrated through a GF/C glass fiber filter type 692 (Whatman). The concentrations of ammonium in the influent for the reactors are presented in Table 1. K_2_HPO_4_ was not used in the SR reactor because it precipitated at high salinity.

**Table 1:** Experimental conditions for freshwater (FR) and seawater (SR) reactors.

Time of operation (days)	Influent NH_4_^+^-N (mg N L^−1^)	HRT (h)	NLR (mg N L^−1^ h^−1^)	Salinity (‰)
Freshwater reactor (FR)				
Phase I				
1–18	40.0 ± 2.0	23.5 ± 3.2	1.7 ± 0.27	0
19–37	109.2 ± 2.4	13.5	8.0 ± 0.19	0
Phase II				
38–62	102.2 ± 1.3	29.3	3.5 ± 0.05	33
63–75	11.0 ± 0.6	14.0	0.8 ± 0.04	33
76–103	11.6 ± 0.5	29.3	0.4 ± 0.01	33
104–127	6.0 ± 0.04	29.3	0.2 ± 0.01	33
Seawater reactor (SR)				
Phase I				
1–18	36.5 ± 4.0	23.4 ± 5.35	1.6 ± 0.54	33
19–37	96.4 ± 7.7	13.0	7.4 ± 0.60	33
Phase II				
38–52	104.0 ± 4.3	13.0	8.1 ± 0.20	0
53–60	31.6	13.0	2.4 ± 0.0	0
61–131	11.7–84.3	12.5 ± 0.95	0.93–7.7	0

*HRT* hydraulic retention time, *NLR* nitrogen loading rate.

### Experimental design

To evaluate the long-term responses of these two nitrifying cultures to the abrupt change in the salinity, the strategy was as follows: In Phase I, the cultures were operated at native salinity and once they reached stability in the ammonium conversion rate, the growth media of the reactors were interchanged (Phase II). The freshwater medium in reactor FR was changed to seawater medium and the seawater medium in reactor SR was changed to freshwater medium. The experimental conditions are presented in Table 1. Before transfer, the acute effect of the salinity changes was evaluated in batch experiments by a capacity test and a toxicity test as described below.

**Figure 1:**
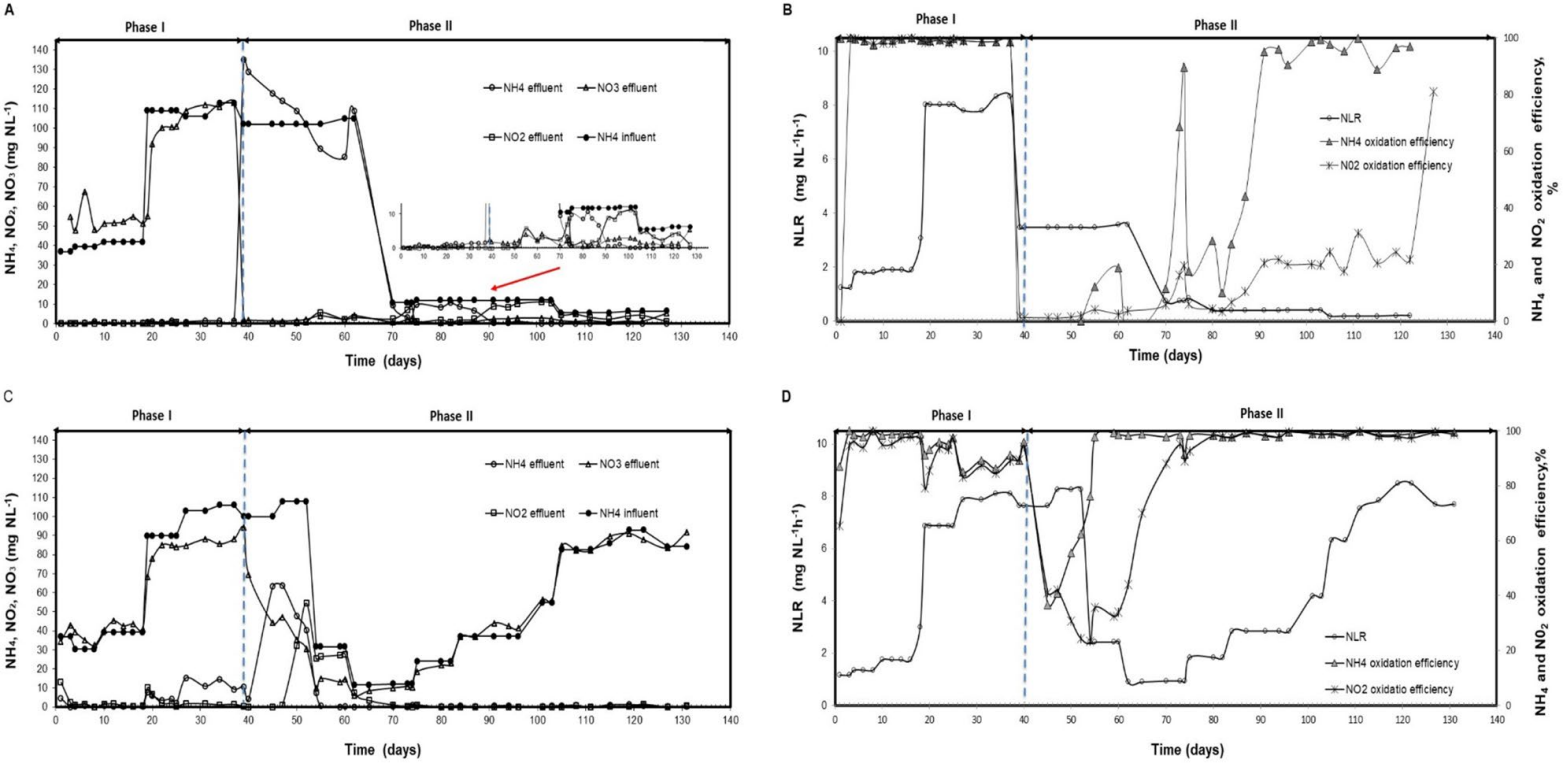
On the left side (**A**,**C**): ammonium concentration in the influent and ammonium, nitrate and nitrite concentrations in the effluent. On the right side (**B**,**D**): nitrogen loading rate (NLR), NH_4_^+^ oxidation rate and NO_2_^−^ oxidation rate during the whole experimental period. Freshwater reactor (FR) represented by A and B. Seawater reactor represented by (**C**) and (**D**). The dotted line indicates the change in salinity.

The experimental set-up in this study was similar to that reported previously^13^. Briefly, two MBB glass reactors with a working volume of 0.7 L (ht: 15 cm, diameter: 9 cm) were inoculated with two different nitrifying cultures: FR reactor with freshwater culture and SR reactor with seawater culture, see the section below, and operated in a continuously fed regime during 127 and 131 days for FR and SR, respectively (Fig. 1 and Table 1). The reactors contained biofilm carriers type Kaldnes K1, surface area: 0.50 m^2^ L^−1^, with a volume fraction of 30% (V_support_/V_reactor_). The dissolved oxygen concentration was kept at 7.5 ± 0.6 mg L^−1^ for the whole period to avoid oxygen limitation of the outer nitrifiers, and the temperature was kept at 25 °C using water-jackets on the reactors connected to a Cole-Parmer Polystat water bath. The pH was maintained between 7.4 and 7.8 by automatic addition of 0.5 M HCL or NaOH. All reactors were mechanically mixed by stirring at 250 rpm.

### Short-term effects of salinity change on nitrification activity

The maximal nitrification activity of the two nitrifying cultures was determined when ammonium conversion stabilized and before the transfer in salinity (i.e. end of Phase I). For this purpose, a capacity test was carried out. The reactors were operated in batch mode and fresh medium with an ammonium concentration of 93 ± 0.4 mg NH_4_-N L^**−**1^ was added at time zero. Samples for analysis of NH_4_^+^–N, NO_3_–N, and NO_2_–N were withdrawn and filtered every 15 min the first half hour and then each 30 min during the next 4 h. The maximum ammonium and nitrite oxidation rates were calculated by linear regression using the concentration versus time data.

To estimate the effect of an abrupt change in the salinity on the maximum nitrifying performance, a toxicity test was carried out. To do so, the freshwater reactor was filled with seawater of salinity 33‰ and the seawater reactor was filled with freshwater base medium free of salt. The starting concentration of ammonium was 107 ± 3.5 mg NH4-NL^−1^. The same variables as for the capacity test were measured at regular time intervals over a period of 13 h. The ammonium and nitrite oxidation rates were calculated as described above and compared with values obtained in the capacity test.

### Sources of biomass and sampling

The freshwater culture was developed from local municipal wastewater (Trondheim, Norway) since 1996 and enriched on purely inorganic media. The seawater culture was developed at NTNU Sealab (Trondheim, Norway), based on moving bed biofilm carriers used in recirculating aquaculture systems (RAS) and fed with ammonia and seawater from approx.70 m depth at a salinity of 33‰ from the Trondheim’s fjord since 2005^13^.

For molecular analysis, samples of biofilm carriers from both reactors were collected throughout the experiment. For the FR reactor, the salinity change was performed at day 39, with samples taken before at days 31 (F5) and 34 (F6), and after change at days 45 (F8), 50 (F9), 55 (F10), 65 (F12), 70 (F13) and 75 (F14). To obtain more samples to evaluate the microbial community at original salinity (freshwater conditions), two samples of biofilm carriers of the same origin collected at days 35 (F3) and 39 (F4) in a previous study carried out at same conditions^13^ were included. For the SR reactor, the salinity shift took place at day 38, and samples were taken before at days 31(S5) and 34(S6), and after salinity change at days 40(S7), 45(S8), 50(S9), 55(S10), 60(S11), 65 (S12), 70(S13) (see Fig. 1B). As for the FR reactor, two more samples collected at days 26 (S3) and 30 (S4) from a previous study carried out at same saline conditions^13^ were included in the total analysis.

### Chemical analysis

Analysis of nitrogen compounds was performed as described in Gonzalez-Silva et al.^13^. Briefly, water samples taken from the reactors were filtered through a 0.45 μm pore size filter. The concentrations of NH_4_^+^–N, NO_3_^−^–N, NO_2_^−^–N were determined on a Lasa 100 photometer (Hach Lange, Germany) using standard Hach Lange cuvette test for each variable. Samples taken from media containing salt were filtered through a Dr. Lange chloride elimination syringe (LCW 925) before analysis. Dissolved oxygen was monitored by an Oxi 3315 hand held digital meter connected to a FDO 925 oxygen electrode (WTW). The pH was measured with a pH electrode connected to a control system (Consort controller R301).

### Molecular biological analyses of bacterial community composition by 454-pyrosequencing

DNA of biofilm carriers was extracted using the Power Soil DNA Isolation Kit from MO BIO Laboratories, by adding the complete carrier into the lysis tubes provided with the kit. The variable region 4 of the bacterial 16S rRNA gene was amplified for each sample by a semi-nested PCR protocol with the primers described in Vik et al.^14^ For the rest, the PCR was performed using the SequalPrep Normalization Plates (96) (Invitrogen) to generate an equimolar amplicon library. The pooled amplicon sample was concentrated using Amicon Ultra 0.5 mL 30 K spin columns (Millipore) and sequenced on ¼ of a 454 plate with a GS FLX instrument at the Norwegian Sequencing Centre (http://www.sequencing.uio.no)^15^. The resulting pyrosequencing data were deposited at the European Nucleotide Archive (study accession number PRJEB12955 and sample accession numbers ERS1076289–ERS1076309.

### Processing of DNA sequences and statistical analysis

Processing sequencing data was analyzed using the Quantitative Insights into Microbial Ecology (QIIME) pipeline version 1.8.0^16^.Sequences were screened for quality; low-quality reads were removed, sequences had a length of at least 200 bp, a minimum quality score of 25, and no ambiguous bases in the primer sequence. The sequences were de-noised using the denoise wrapper.py script in Qiime. UCHIME algorithm was used to detect possible chimeric sequences, which were removed from the dataset^17^. OTU picking and taxonomic assignments was performed using the open-reference OTU picking workflow in Qiime with the Greengenes core dataset gg_13_5.fasta. The dataset was examined for the know AOB, NOB and potential heterotrophic bacteria as described in Gonzalez-Silva et al.^13^ and the relative abundances were determined as % of the total reads for each sample.

Paleontological statistics software package (PAST) version 2.17c was used to carry out all statistical analyses^18^. The diversity indices richness and Shannon’s^19^ were calculated. Ordination based on Bray–Curtis similarity coefficient was done with Principal coordinate analysis (PCoA)^20^. Tests of significant differences in community structure between groups of samples were done by Non-parametric Multivariate Analysis of Variance (PERMANOVA) using Bray–Curtis and Jaccard similarities^21^. The Similarity Percentages (SIMPER) analysis^22^ was performed to determine the contribution of individual AOB and NOB OTUs of the nitrifying community to the Bray–Curtis dissimilarity between the samples of the FR and SR reactors. Venn diagrams were constructed to compare the distribution of unique and shared AOB and NOB OTUs among different conditions.

Phylogenetic trees were constructed using MEGA 4.0^23^ applying the neighbor-joining method and bootstrap consensus tree inferred from 1000 replicates. Evolutionary distances were computed using the Maximum composite likelihood method, given in the units of number of base substitutions per site. The sequences representing previously identified AOB and NOB bacteria were downloaded from the Ribosomal Database Project (http://rdp.cme.msu.edu/) using the parameters “isolates” with size ≥ 1200 nucleotides, and “good quality”.

### Ethical approval

This article does not contain any studies with human participants or animals performed by any of the authors.

## Results

### Performance of continuous reactors and batch tests

#### FR reactor

In Phase I (days 1 to 37), the FR reactor was adjusted by one increase in the nitrogen loading rate (NLR), from 1.7 ± 0.2 to 8.0 ± 0.1 mg N L^**−**1^ h^**−**1^ (Table 1 and Fig. 1B). During this period the FR culture responded well to increases in NLR achieving an average ammonium oxidation efficiency of 91 ± 0.6% (Fig. 1A,B).

A capacity test in Phase I indicated maximal ammonium and nitrite oxidation rates of 11.9 ± 0.3 and 12.0 ± 0.3 mg N L^−1^ h^−1^, respectively (Fig. S1A; Supplemental Material and “Short-term effects of salinity change on nitrification activity” section ), which was 50% higher than the rate under operating conditions. However, the shock loading toxicity test at 33‰ salinity resulted in complete loss of nitrifying activity (Fig. S1B; Supplemental Material and “Short-term effects of salinity change on nitrification activity”section ).

During Phase II (days 39–127) at 33‰ salinity, the salinity increase caused serious inhibition of the nitrifying bacteria (Fig. 1A,B). Calculations of free ammonia and nitrous acid concentrations (days 39–52 of Phase II), using the relationship from Anthonisen et al.^24^, were in the range of 1.5 to 5 mg L^−1^ and < 1 × 10^−6^ mg L^−1^, respectively. To enhance the nitrifying activity and minimize inhibition of ammonia and nitrite, the NLR was stepwise decreased, first from 8 to 0.4 mg N L^−1^ h^−1^ and then 0.2 mg N L^−1^ d^−1^ at day 104 (Table 1 and Fig. 1B). From days 91 to 122 some NOB activity was observed (Fig. 1B). In conclusion, the nitrifying activity in FR was not significantly recovered within 90 days after the cross-transfer due to the low capacity of NOBs.

#### SR reactor

In Phase I, the SR reactor showed some accumulation of nitrite and ammonium (days 10–20 and 27–37, respectively), probably due to a transient effect when the NLR was increased (Fig. 1C). However, the average ammonium and nitrite removal efficiency during this period was satisfactory (94 ± 5.0 and 90.7 ± 8.3%, respectively; Fig. 1D).

The capacity test at the end of Phase I indicated maximum ammonium and nitrite oxidation rates of 11.4 ± 0.7 and 11.3 ± 0.6 mg N L^−1^ h^−1^, respectively, (Fig. S1C; Supplemental Material and “Short-term effects of salinity change on nitrification activity”section ), which are 50% higher than under operation conditions. However, the toxicity test indicated a 30% reduction in the nitrification rates after exposure to a medium in low salt (Fig. S1D; Supplemental Material and “Short-term effects of salinity change on nitrification activity”section ).

In the initial part of Phase II, the nitrifying rate was reduced significantly, with an accumulation of ammonium and nitrite (days 45–52 and 50–60, respectively) in the effluent (Fig. 1C). Calculations of free ammonia and nitric acid during this build-up period were in the range of 1.4–2.5 mg L^−1^ and < 4.5 × 10^−6^ mg L^−1^, respectively. To enhance the performance and minimize possible inhibition due to ammonium, free ammonia, and nitrite, the NLR was decreased, first from 8.1 to 2.4 mg N L^−1^ d^−1^ at day 53, and then to 0.93 at day 61 (Table 1 and Fig. 1D). From day 61 the nitrite level was decreasing (Fig. 1D) and therefore the NLR was increased stepwise from 0.93 to 7.3 mg N L^−1^ h^−1^ (Table1 and Fig. 1D). The nitrifying performance responded well to those increases, achieving average ammonium and nitrite oxidation efficiencies of 97.8 ± 2.5% and 97.12 ± 12.3%, respectively, from day 73 to the end. In conclusion, the nitrifying activity of SR reactor was completely recovered after 70 days of operation under freshwater conditions (Fig. 1D).

### Bacterial community structure based on 16S rRNA gene amplicons

**Figure 2:**
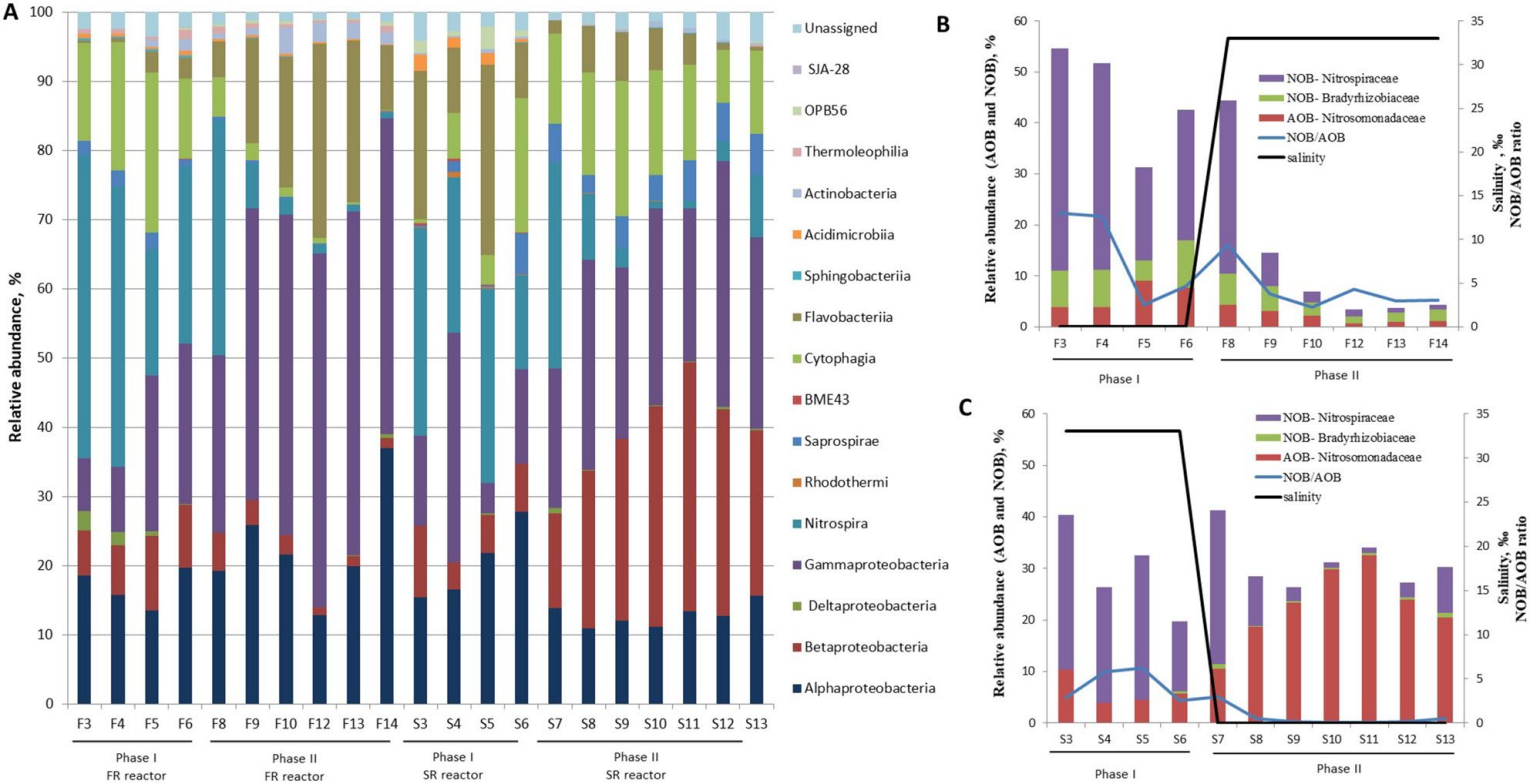
(**A**) Relative abundance per sample of the different classes in the samples. For freshwater reactor (FR), F3–F14 samples in phase I and II with freshwater and seawater environment, respectively. For seawater reactor (SR), S3–S13 samples in phase I and II with seawater and freshwater environment, respectively. (**B**,**C**) Average relative abundances at family level of the AOB and NOB guilds, the NOB/AOB ratio and the salinity for FR reactor (**B**) and SR reactor (**C**).

Pyrosequencing yielded a total of 1200 OTUs and 91,380 reads for all samples collected in the FR and SR reactor, after removing singletons. The number of bacterial phyla detected in the reactors was 19 and 21 for FR and SR, respectively (Fig. 2A). The four most abundant phyla in both reactors were *Proteobacteria* (average relative abundance 58.1 ± 15.0%), *Bacteroidetes* (22.0 ± 7.0%), *Nitrospirae* (15.4 ± 14.0%) and *Actinobacteria* (1.8 ± 1.0%).

Approximately 90% of the OTUs detected in the data set did not represent nitrifiers and were classified as potential heterotrophic bacteria. The average relative abundance of these heterotrophs in FR accounted for 55 and 95% of the total reads in Phase I and II respectively, whereas for SR it was 70% of total reads in both Phases I and II (Fig. 2A). *Alpha* and *Gamma-proteobacteria* were the dominating classes for the heterotrophs under both freshwater and seawater conditions. *Cytophagia* was more abundant at freshwater conditions and *Flavobacteriia* at seawater conditions.

Of the total OTUs, 116 were classified as nitrifying bacteria. The AOB guild was composed of 49 OTUs (all classified as *Nitrosomonadaceae*), while 67 OTUs belonged to the NOB guild (27 classified as *Bradyrhizobiaceae*, 40 as *Nitrospiraceae*). The AOB and NOB guilds in the FR represented 4 and 23% of the total reads, respectively. However, the average relative abundance of the nitrifying community decreased by 90% after transfer to marine salinity (Fig. 2B). In Phase I of FR the NOB/AOB ratio was 8.4, whereas in Phase II it decreased to 3.2. The relative abundance of the AOB and NOB guilds were 4 and 12 times lower, respectively, compared to the freshwater conditions of Phase I (Fig. 2B). In the SR, 18 and 13% of the total reads belonged to the AOB and the NOB guild, respectively, and the nitrifying community remained at 30% after the change to freshwater (Fig. 2C). In Phase I of SR, the average NOB/AOB ratio was 4.0, whereas in Phase II this ratio was 25 times lower. The average relative abundances of the AOB and NOB guilds were 4 times higher and 6 times lower respectively, compared to the marine salinity of Phase I (Fig. 2C). It is interesting to notice that the most dominant NOB species for FR (Phase I) and SR (Phase I and II) was *Nitrospira*, which belongs to the family *Nitrospiraceae*^25^, (Fig. 2B,C). The analysis of the trending up or down of AOB, NOB, and NOB/AOB ratio of FR and SR samples (Fig. 2B,C) by linear regression were statistically significant with *p* values < 0.01.

**Figure 3:**
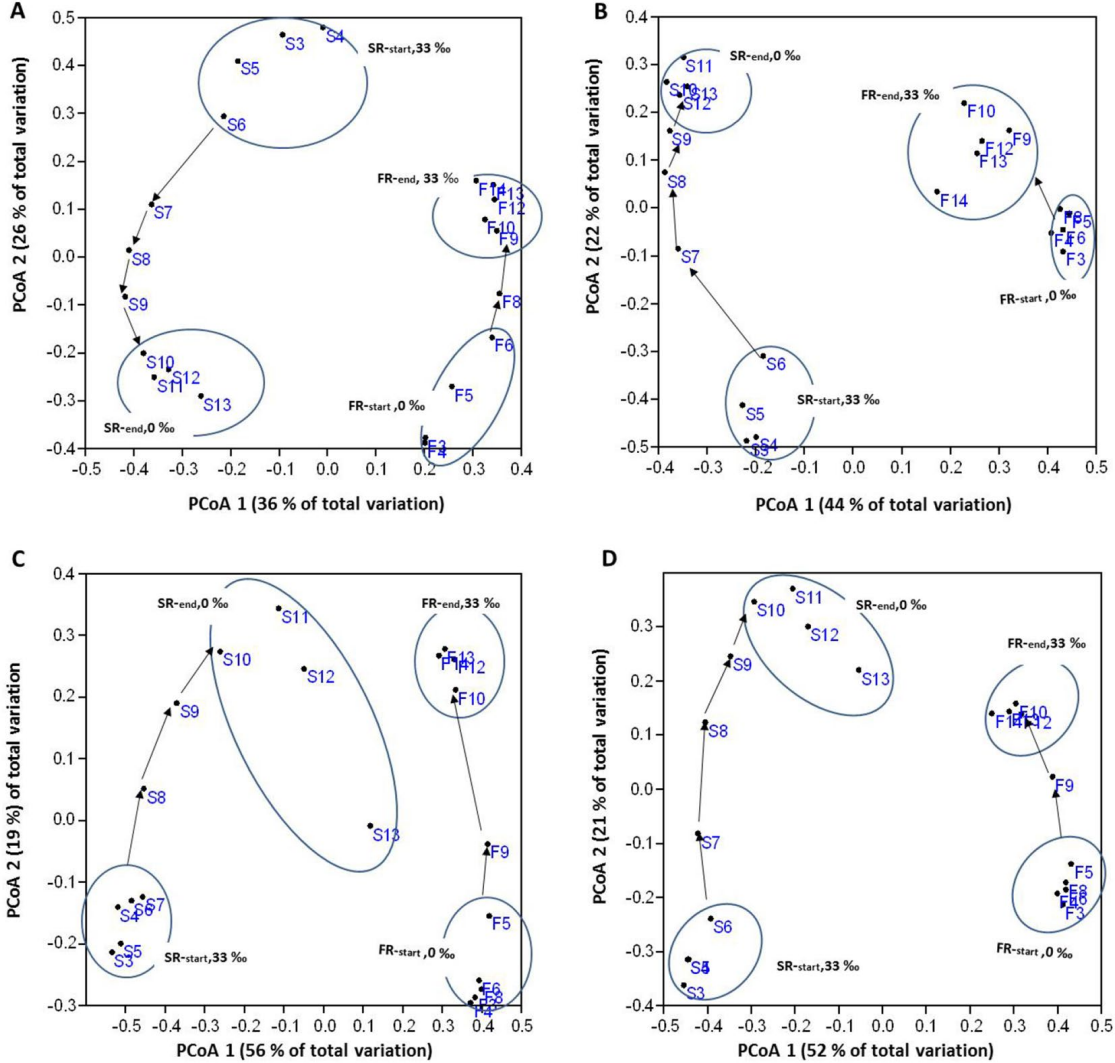
Principal coordinates analysis (PCoA) based on Bray–Curtis similarity of pyrosequencing data. F4–F14: samples from the freshwater reactor (FR); S3–S13: samples from seawater reactor (SR). (**A**) Based on total OTUs; (**B**) Based only on OTUs representing the AOB guild; (**C**) Based on OTUs representing the NOB guild, and (**D**) Based on OTUs representing the nitrifying community (AOB plus NOB guilds). Four groups of samples are identified FR_start_, FR_end_, SR_start,_ and SR_end_. The arrows between each group show the time line for sampling.

The changes in the microbial communities of the FR and SR reactors before and after the changes in salinity were evaluated by a PCoA based on Bray–Curtis similarities, for the total community, the AOB guild, the NOB guild and the nitrifying community (AOB + NOB) (Fig. 3). When the salinity was changed, the similarities of the FR and SR samples compared to Phase I gradually decreased as a function of time, indicating a salinity induced succession of the microbial communities (Fig. 3). The PCoA axis 1 seems to be related to the salinity by separating SR and FR samples, whereas the PCoA axis 2 seems to reflect the succession of the microbial communities. As shown in Fig. 3, four different clusters were identified and named FR-start, FR-end, SR-start, and SR-end. For the total community, including all OTUs (Fig. 3A), we observed that the succession of FR and SR samples were going in an opposite direction largely parallel to PCoA 2, whereas, for the nitrifying community and AOB and NOB guilds, the succession of both reactors were in the same direction (Fig. 3B–D). One-way PERMANOVA test based on Bray–Curtis similarity confirmed significant differences in the four clusters of all these communities (*p* < 0.03). Significant differences in species inventory were also confirmed based on Jaccard similarity (*p* < 0.04), indicating a difference in both species inventory and abundance. These four clusters form the basis of the subsequent exploration.

**Figure 4:**
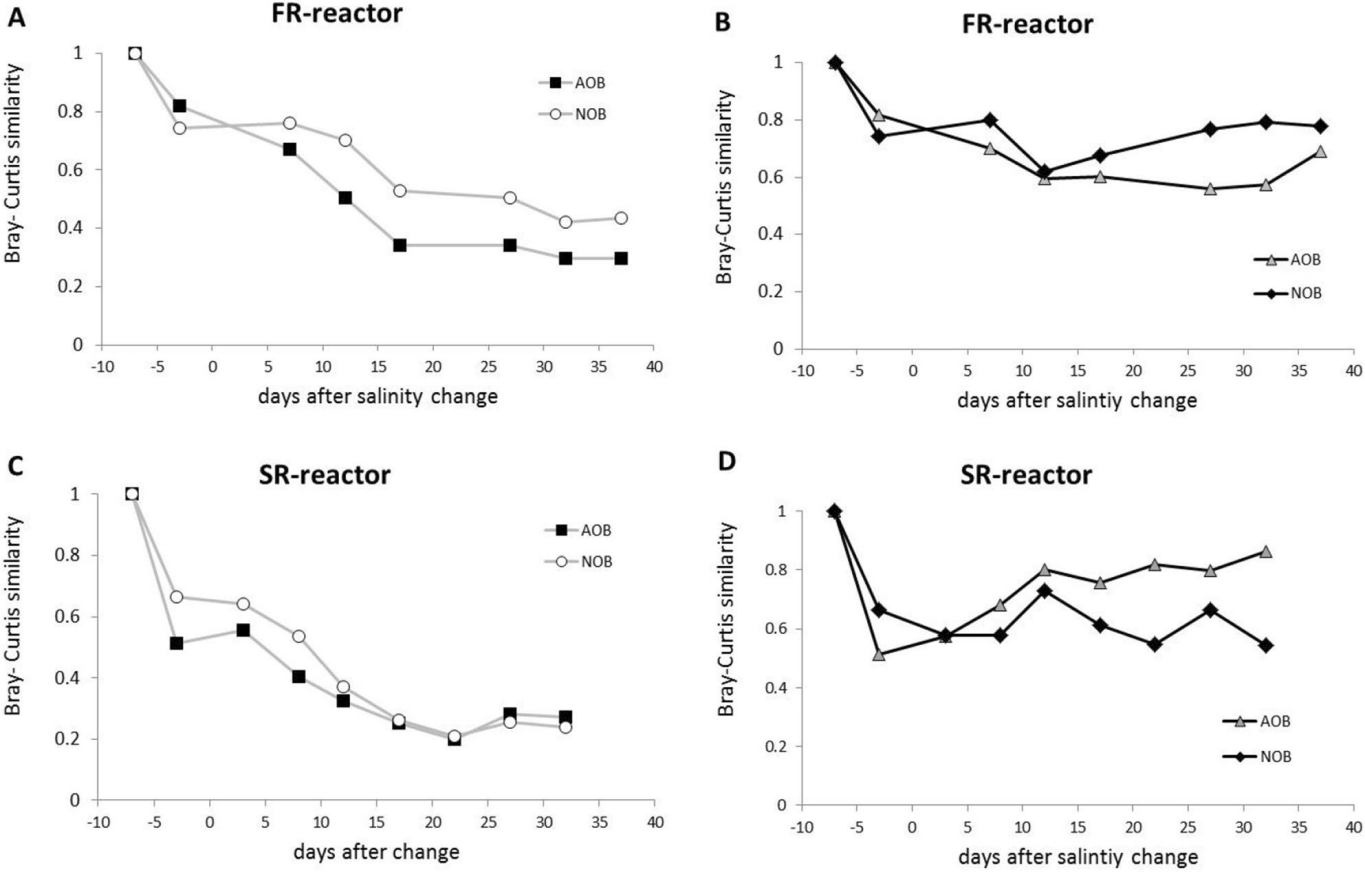
Succession of the microbial community. (**A**) Bray–Curtis similarity between all samples versus the initial F5 sample in the FR reactor. (**B**) Bray–curtis similarity based on comparison with previous/next sample in time for the FR reactor. (**C**) Similar to panel (**A**) but for SR reactor using S5 sample as reference. (**D**) Similar to panel (**B**) but for the SR reactor.

The succession of the nitrifying communities of FR and SR reactors were then analyzed independently for the AOB (Fig. 3B) and NOB (Fig. 3C) guilds by two different approaches. First, the Bray–Curtis similarities were determined for each sample compared to the first sample taken for each reactor (Fig. 4A,C). Second, the Bray–Curtis similarities were determined for comparisons of each SR and FR samples to the preceding sample in the same reactor (Fig. 4B,D). This indicates gradual changes in the microbial communities of both reactors, reaching apparent stability toward the end of sampling (Fig. 4A,C). The analysis of the successions of AOB and NOB based on Bray–Curtis similarity by linear regression indicated that were statistically significant with *p* values < 0.03. However, only the NOB guild of the FR reactor and AOB guild of the SR reactor stabilized at the end of sampling (high Bray–Curtis similarity, 0.78–0.86; Fig. 4B,D).

**Figure 5:**
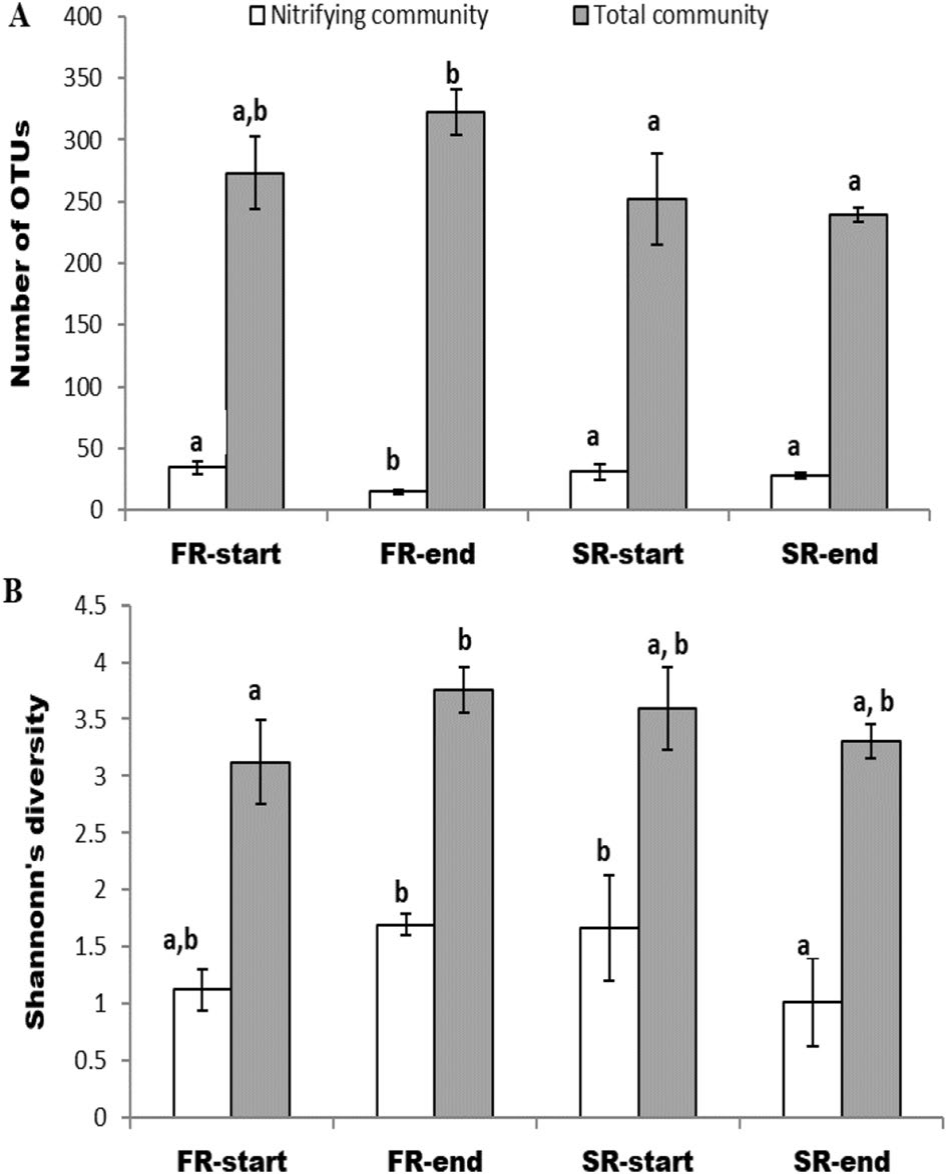
Diversity indices based on the pyrosequencing data of the total and the nitrifying communities. (**A**) Richness (observed OTUs). (**B**) Shannon’s diversity index. For freshwater reactor (FR): F3–F6 (FR_start_) and F9–F14 (FR_end_). For seawater reactor (SR): S3–S6 (SR_start_), and S10–S13 (SR_end_). Equal letters (a, b) above bars indicate no significant difference (*p* > 0.05) and different letters (a, b) indicate significant statistical differences (*p* < 0.05) by ANOVA and Tukey’s multiple comparisons. Error bars show standard error of the mean.

For the total community, the average OTU richness for the samples of both the SR-start and end were significantly lower than for the FR-end samples, whereas for the nitrifying community the OTU richness for the samples of the FR-end was significantly lower than for the other three groups (Fig. 5A). The Shannon’s diversity index for the total community was significantly higher for the samples of the FR-end than for the FR-start, whereas Shannon’s diversity index in the nitrifying community was highest for the samples of the FR-end and SR-start (Fig. 5B). The average Bray–Curtis similarity calculated within each of the four clusters of nitrifying communities was high (0.6–0.75), whereas the similarities were low for comparisons of the samples between the –end and –start clusters (0.36–0.24), indicating large changes in the nitrifying communities after salinity change in both reactors (Fig. S3; Supplemental Material).

SIMPER analysis showed that the 10 most significant OTUs contributed 92.7% of the total difference between the community clusters (Table 2). The 4 most influential of these OTUs contributed 75% of this difference. The relative abundance of these 10 OTUs in each cluster is summarized in Table 2. A phylogenetic analysis (Fig. S2, Supplemental material) identified the closest relatives of these 10 AOB and NOB OTUs (Table 2). The largest change in abundance of the most dominant AOB and NOB OTUs was observed in the SR-start and SR-end clusters, with a dramatic increase in abundance of an OTU related to *Nitrosoccus mobilis*. Interestingly, the abundance of this OTU also increased in the FR reactor. In FR, the abundance of an OTU (1124569) representing *Nitrospira* decreased from 67 (FR-start) to 24% (FR-end), while the abundance of a *Nitrobacter* OTU (220928) increased three-fold.

**Table 2:** Results from SIMPER (similarity percentage) analysis showing the ten most significant OTUs contributing to the total difference between the four groups of samples in FR and SR reactors and the contribution of these OTUs to the difference between the four groups. Also included are the results from the classification of these OTUs based on QIIME pipeline and Greengenes, and phylogenetic analysis.

OTU ID	SIMPER analysis results		Guild/(Classification (lowest taxonomic level)	Phylogenetic analysis (Maximum Composite likelihood method/Ribosomal Database Project)	Average relative abundance ± SD per sample of nitrifying reads (%)			
					FR-samples		SR-samples	
	Contribution%	Cumulative%	Greengenes		FR-start (0‰)	FR-end (33‰)	SR-start (33‰)	SR-end (0‰)
1124569	29.1	29.1	NOB/*Nitrospiraceae*	*Candidatus Nitrospira*	66.8 ± 8.7	24.0 ± 6.9	0.0 ± 0.0	6.3 ± 9.3
18274	23.8	52.9	AOB/*Nitrosomonadaceae*	*Nitrosococcus mobilis*	0.2 ± 0.2	12.2 ± 1.7	0.3 ± 0.5	77.8 ± 13.4
758679	15.4	68.3	NOB/*Nitrospiraceae*	*Candidatus Nitrospira salsa*	0.0 ± 0.0	0.2 ± 0.2	51.0 ± 18.5	0.9 ± 0.8
220928	6.5	74.9	NOB/*Bradyrhizobiaceae*	*Nitrobacter sp.*	13.8 ± 3.2	42.0 ± 4.7	0.1 ± 0.2	1.2 ± 0.9
6540	5.3	80.2	AOB/*Nitrosomonadaceae*	*Nitrosomonas* sp.	12.9 ± 8.0	7.2 ± 1.9	0.1 ± 0.0	0.0 ± 0.0
27445	4.4	84.7	AOB/*Nitrosomonadaceae*	*Nitrosomonas aestuarii*	0.0 ± 0.0	2.0 ± 3.2	14.8 ± 4.3	0.9 ± 0.5
345421	3.4	88.1	NOB/*Nitrospiraceae*	*Nitrospira marina*	0.0 ± 0.0	0.0 ± 0.0	10.9 ± 4.2	1.7 ± 1.0
1443	2.5	90.7	NOB/*Nitrospiraceae*	*Nitrospira marina*	0.0 ± 0.0	0.0 ± 0.0	8.3 ± 6.0	1.6 ± 2.3
265873	1.1	91.8	AOB/*Nitrosomonadaceae*	*Nitrosomonas cryotolerans*	0.0 ± 0.0	0.0 ± 0.0	2.8 ± 1.6	1.52 ± 0.6
2709955	0.9	92.7	NOB/*Nitrospiraceae*	*Nitrospira marina*	0.0 ± 0.0	0.0 ± 0.0	3.2 ± 0.9	0.0 ± 0.0

**Figure 6:**
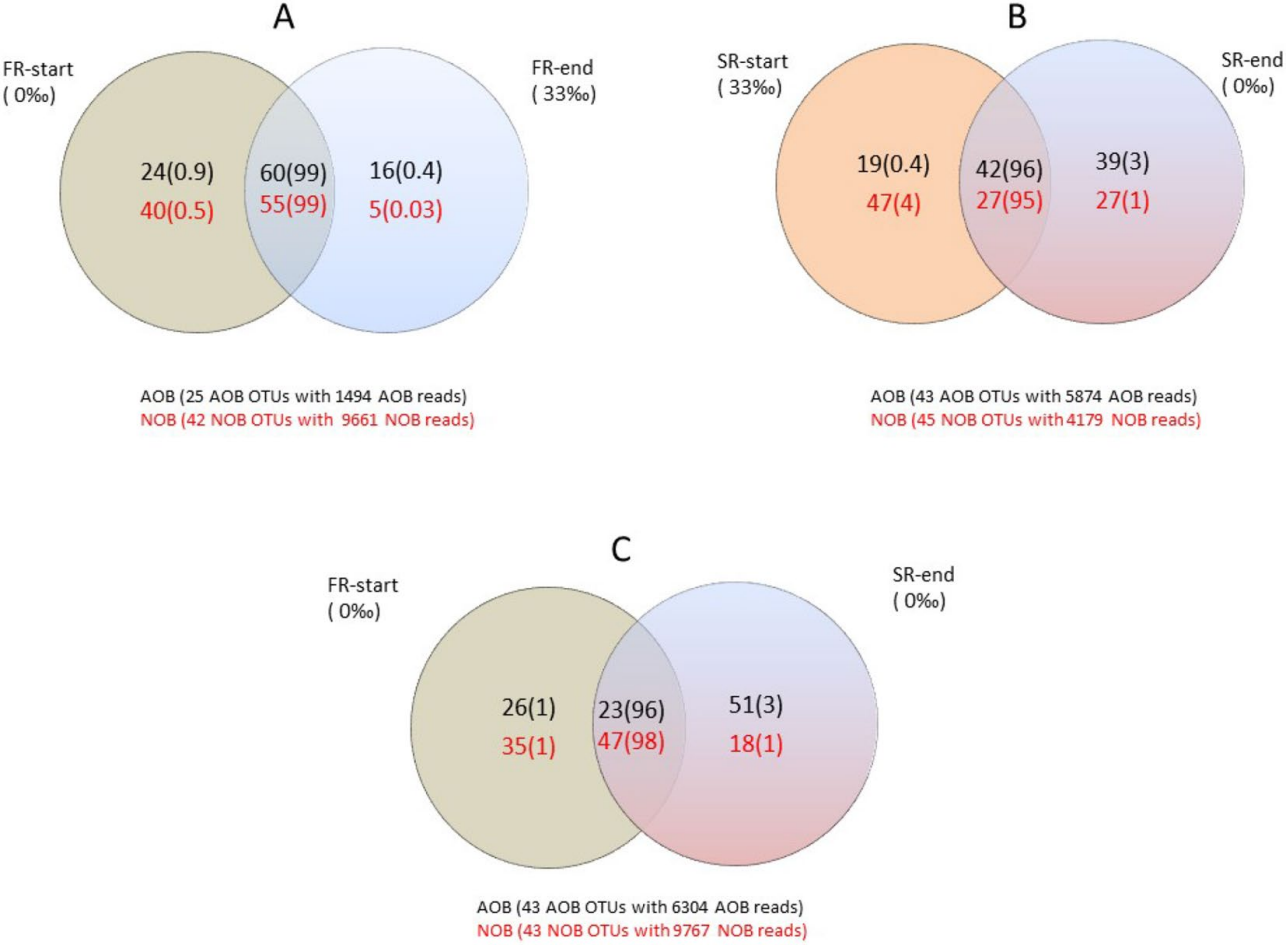
Venn diagrams in percentages showing the number of unique and shared OTUs, representing nitrifying bacteria for FR-start versus FR-end of freshwater reactor (**A**), SR-start versus SR-end of seawater reactor (**B**), at freshwater environment : FR-start vs SR-end (**C**). The numbers in black and red colour in each group indicate the number of unique or shared OTUs for the AOB and NOB guilds, respectively. The value in parenthesis shows the percentage of these OTUs represent of the total AOB or NOB sequences reads (e.g. for the Panel (**A**): 24 unique AOBs constitute 0.9% of the total 1494 AOB reads). Numbers in overlapping regions indicate number of shared OTU's.

Venn diagrams were constructed to find the number of shared and unique OTUs among the groups (Fig. 6). We observed that both the fraction of shared OTUs and the abundance of those OTUs were important for explaining the differences between the groups. In most of the cases, the shared OTUs were highly abundant only in one of the groups (Table 2). For the FR-start and end, the Venn diagram indicated that these clusters shared 60 and 55% of AOB and NOB OTUs, respectively, of the total found in both clusters (Fig. 6A). However, only the AOB OTU 6540, closely related with *Nitrosomonas* sp., and the NOB OTUs 1124569 and 220928, closely related with *Ca. Nitrospira* and *Nitrobacter* sp., respectively, where highly abundant in both clusters (Table 2). The SR groups, on the other hand, shared 42 and 27% of AOB and NOB OTUs, respectively, of the total found in both groups (Fig. 6B). However, different OTUs dominated by both groups. The SR-start was dominated by OTUs closely related to *Nitrosomonas aestuarii* and *Ca. Nitrospira salsa* (OTUs 27,445 and OTU758679; Table 2), whereas SR-end by OTUs closely related to *Nitrosococus mobilis* and Ca. *Nitrospira*, respectively, (OTUs 18274 and 1124569; Table 2).

FR-start and SR-end samples represent two well-functioning cultures at freshwater conditions, but with different adaptation regime and inoculum (see “Sources of biomass and sampling” section). The FR-start samples were enriched in a freshwater environment for several years, see “Sources of biomass and sampling” section, whereas the SR-end samples were exposed to freshwater less than 3 months (Fig. 1C). The average Bray–Curtis similarity among these samples was 0.19, indicating low similarity in community structure (Fig. S3; Supplemental Material). However, Venn diagram indicates that these samples share 23 and 47% of AOB and NOB OTUs, respectively (Fig. 6C). The most abundant shared OTUs among these groups were the four shown in Table 2. In the AOB guild, the OTU 6540, closely related with *Nitrosomonas* sp., was highly abundant in FR-start, whereas the OTU18274, closely related with *N. mobilis*, was highly abundant in SR-end. In the NOB guild, the OTUs 1124569 closely related to *Ca. Nitrospira* was highly abundant in FR-start sample (Table 2).

## Discussion

### Physiological responses to salinity stress (FR and SR reactors)

Moderate to high salinities are known to produce inhibitory or toxic effects on bacteria not adapted to high salinity, and high salt concentrations (> 10‰) have been shown to cause loss of activity of cells^26^. This may explain the poor nitrifying activity of the freshwater culture in FR reactor after the abrupt change to marine salinity (Phase II). It has been reported that AOB shows an inhibition threshold for free ammonia between 10 and 150 mg L^**−**1^^24^. Determination of free ammonia during the initial Phase II of this study was in the range of 1.5 to 5 mg L^**−**1^, therefore the free ammonia generated did not have any inhibitory effects on AOB and salinity was the only factor that negatively affected the ammonia oxidation efficiency (Fig. 1B). After the salinity changes (Phase II), the AOBs seemed to be less sensitive than the NOBs, because we observed better AOB than NOB activity (Fig. 1B). This is in agreement with previous studies, reporting that NOBs are more susceptible to increasing salt levels than AOBs^27–31^, probably because salinity, in particular, imposes thermodynamic constraints for chemolithoautotrophic NOB, as less energy is gained from their metabolism to support both growth and osmoregulation^32,33^.

The SR culture responded better to freshwater conditions than the FR to seawater. The AOB guild of the SR reactor only needed seven days (from days 38–45; Fig. 1D) to recover its AOB activity to the same NLR in freshwater as in Phase I, reaching 50% ammonium oxidation efficiency 11 days after the salinity change (Phase II; Fig. 1D). This response may be explained by the fourfold increase in abundance of AOB in Phase II compared with Phase I. The NOB guild, on the other hand, showed a lag phase of approximately 15 days, before the nitrite oxidation increased rapidly when the NLR was first decreased and then stepwise increased again (Fig. 1D and Table 1). In addition to the salinity change, this lag phase might also be related to a temporal NOB inhibition caused by the potential presence of the free ammonia (1.4–2.5 mg L^−1^) generated during ammonium accumulation in Phase II (Fig. 1D).

In this study, all samples of both FR and SR in Phase I presented a higher abundance of NOB than AOB. The classical concept of nitrification describes a mutualistic symbiosis where NOBs depend on nitrite produced by AOBs, which benefit from nitrite detoxification by NOB^34^. Then the ratio of AOB to NOB should reflect the availability of energy from the respective metabolic reactions of these two ecological niches^35^.Thermodynamic calculations suggest that AOB should outnumber NOBs because the energy available from the oxidation of ammonia to nitrite (∆G°′ =  − 275 kJ mol^−1^) is greater than the amount of energy available from the oxidation of nitrite to nitrate ( ∆G°′ =  − 76 kJ mol^−1^)^35^. However, this is contradictory to the imbalances in abundances of AOB and NOB observed in this study, where the samples of both FR and SR in Phase I presented a higher relative abundance of *Nitrospira* than AOBs. Imbalances in abundances of nitrite- and ammonia-oxidizing bacteria have also been observed in previous studies^36,37^. Several authors have reported that high abundances of *Nitrospira*, which exceed the amounts of AOB and ammonia-oxidizing archaea (AOA) in the same habitat, can indicate the presence of complete ammonia oxidizers, comammox,^34,35,38,39^. However, in this study more research is needed to prove the presence of comammox in FR and SR reactors.

On the other hand, it has been reported that gradual reduction of salinity for nitrifiers enriched from marine sediments (3.5% salinity ), could be functional if the salinity was changed from 3.5% to no lower than 0.5%^40^. The authors reported that the AOB guild was most affected and that ammonia began to accumulate at very low salt, (0.034%), whereas nitrite only occurred transiently. In the present study, we observed that the NOB guild in the marine culture of SR was more affected by the transition to low salt, but its activity recovered later. This marine culture was robust to the long-term salinity stress introduced by cross-transfer in salinity, showing high resistance in the freshwater environment and achieved satisfactory ammonium and nitrite oxidation efficiencies after 35 days of exposure to freshwater (Fig. 2C). In our previous study, it was observed that this marine culture as well as a brackish culture (at 20‰ salinity) showed high tolerance to freshwater during an acute toxicity test of 3 h^13^. A potential limitation of this study is the fact that the physiological responses to salinity stress of FR and SR reactors cannot be assessed by statistical methods because of the lack of replicates in the experimental design.

Salinity in industrial wastewaters may be highly variable, and fluctuating salt concentrations may create transient shocks^41^. The use of specialized organisms such as halophiles may be a way to enhance the biological treatment of saline wastewaters^42^. Therefore, salt-tolerant cultures, such as the SR culture described in this study, may be suitable and robust inocula for treatment of nitrogen in RAS when fast salinity change is needed in the life cycle of the reared fish, e.g. from hatching to post-smolt in Atlantic salmon production^10^ or in wastewaters with fluctuating salinity, a scenario likely to happen in many industrial effluents^42^.The reason for this was the dominance of species with special physiology (see below).

### Long term community changes after cross-transfer

*Nitrosococcus mobilis*-lineage is reported to be opportunistic *r*-strategists with a relatively high growth rate^43^, and considered to be a salt-resistant nitrifier^44^. Its presence has been reported in brackish water^45^ as well as higher salt levels^12^. This confirms the dominance of this species in seawater in the FR-end (33‰) cluster (OTU 18274; Table 2). However, this species was also the dominant AOB in the SR-end cluster at 0‰ salinity, contradictory to the reports above. Issihiki et at.^46^ reported *N. mobilis* as a high-stress tolerance AOB that can adapt to an unfavorable environment. We observed that *N. mobilis* holds high physiological plasticity towards salinity because it not only tolerated the stress in connection with the abrupt salinity changes but also was able to adapt to the new environment, increasing the relative abundance at seawater and freshwater conditions (OTU 18274; Table 2). *Nitrosomonas* sp., on the other hand, has been reported to adapt to salt levels up to 33 g L^**−**1^ NaCl^28^. These findings are in agreement with our observations because *Nitrosomonas* (OTU 6540) was also present in the FR-end cluster (Table 2).

*Nitrobacter* has been reported as ***r***-strategist when substrates (oxygen and NO_2_^−^) are abundant^47^. This genus has mechanisms to manage osmotic stress and to survive in the marine environment^48^ and has been reported as the only NOB at high salinity^12^. In this study, we observed that the relative abundance of *Nitrobacter* sp. increased in both FR- and SR-end, compared to FR- and SR-start clusters (Table 2). Probably this species was contributing the most to the observed nitrite oxidation in Phase II of the FR reactor (Fig. 1B) because it was the most dominant NOB in FR-end samples (Table 2). *Ca. Nitrospira*, on the other hand, is reported to be K-strategist with a relatively low maximal growth rate^43^. In our study, we observed that this genus (OTU 1124569) holds physiological plasticity towards salinity, with significant survival at high salinity (FR-end; Table 2) and low salinity (SR-end; Table 2).

A major change in species composition was observed in the SR reactor after salinity change. The most dominating AOB and NOB in the seawater SR culture before transfer (S-start), closely related to *Ca. Nitrospira salsa* and *N. aestuarii*, were not competitive at freshwater conditions, and its abundance was drastically decreased in the SR-end cluster (Table 2). These have been described as common species in marine environments^7,49^ and may have an obligate requirement for some salt, e.g. *N. aestuarii* have an optimal growth rate at 1.7%^40,50^. However, the OTU closely related to *Nitrospira marina* and assumed to be a marine species^49,51^ was the second most abundant NOB in both SR clusters (Table 2), indicating a high resistance to survive in freshwater. The presence of this species has been also reported for freshwater aquaria^52^.

It is interesting to compare the freshwater communities of FR-start and SR-end samples (Table 2), both functioning under freshwater conditions. We observed that the NOB abundance of the FR-start and AOB abundance of SR-end accounted for 80% of the nitrifying community of each cluster. *Nitrobacter* has been reported as the sole nitrite oxidizer (NOB) in a well-established freshwater nitrifying reactor^53^. However, in this study, its presence was observed in FR-start and SR-end clusters. This may reflect that this species may play important roles in the nitrite oxidation activity in freshwater conditions.

The main objective of the present work was to evaluate the effect of an abrupt change in salinity of freshwater and seawater nitrifying microbial communities that were long term adapted (years) to their native salinity. This is particularly important issue since salinity has been reported as a strong environmental factor influencing the microbial structure^54^, regulating and reducing bacterial diversity^12,55^, as well as controlling AOB diversity and distribution^56^. Besides, it has been reported that salinity changes also induces changes in the dominant species of AOBs^44^. Our findings contradict the current perspective of the significance of salinity on the structure of nitrifying communities. We observed that independent of salinity some nitrifiers such as *N. mobilis*, *Ca. Nitrospira* and *Nitrobacter* were very successful at both zero and full seawater salinity (Table 2). Besides, we observed the presence of shared AOB and NOB OTUs among FR and SR samples (Venn diagrams, Fig. 6). All these findings contradict the general assumption that salinity is a strong selective force of the nitrifying communities.

## Conclusions

- In this research, we observed that both succession and physiological plasticity were the main mechanisms of the long-term adaption of the nitrifying communities exposed to abrupt salinity changes. However, the strength of these mechanisms was different at the community and OTU levels. Succession of the nitrifying community was the principal mechanism observed at the community level during the cross-transfer between freshwater and seawater, whereas physiological plasticity towards salinity was directly observed at the OTU level, and was present in only a few OTUs such *as N. mobilis*, *Ca. Nitrospira* and *Nitrobacter.*
- Our findings contradict the general assumption that salinity is a strong selective force on the structure of nitrifying communities.
- The seawater culture showed high resistance to stress caused by low-salt and can be a suitable inoculum for ammonium removal from RAS and industrial wastewaters with variable and fast salinity changes. This is due to the presence of species with special physiology such as *Nitrosococcus mobilis* and *Ca. Nitrospira*.
- The results obtained in our work might provide clues for the development of robust nitrifying biofilters in RAS for the production of commercial species such as Atlantic salmon.

## Supplementary Information

Supplementary Information
