# Supplementary material for: Understanding structure/function relationships in nitrifying microbial communities after cross-transfer between freshwater and seawater
Source: Sci Rep. 2021 Feb 3;11:2979. doi: 10.1038/s41598-021-82272-7 (PMC7859187; doi:10.1038/s41598-021-82272-7)
Supplement: Supplementary file 1 — Supplementary Information [file 41598_2021_82272_MOESM1_ESM.docx]

**Supplementary Information**

**Understanding structure/function relationships in nitrifying microbial communities after cross-transfer between freshwater and seawater**

Blanca M. Gonzalez-Silva*^1^, Kjell Rune Jonassen^2^, Ingrid Bakke, Kjetill Østgaard, Olav Vadstein

Department of Biotechnology, Faculty of Natural Sciences and Technology, NTNU Norwegian University of Science and Technology, Sem Saelands v. 6/8, N-7491 Trondheim, Norway.

^1^Present address: Department of Hydraulic and Environmental Engineering, NTNU Norwegian University of Science and Technology, S. P. Andersens veg 5, 7491 Trondheim, Norway

^2^Present address: VEAS, Bjerkåsholmen 125, 3470 Slemmestad Oslo, Norway.

*Address correspondence to Blanca M. Gonzalez Silva, [blanca.g.silva@ntnu.no](mailto:blanca.g.silva@ntnu.no), tel. (+47)41449643,


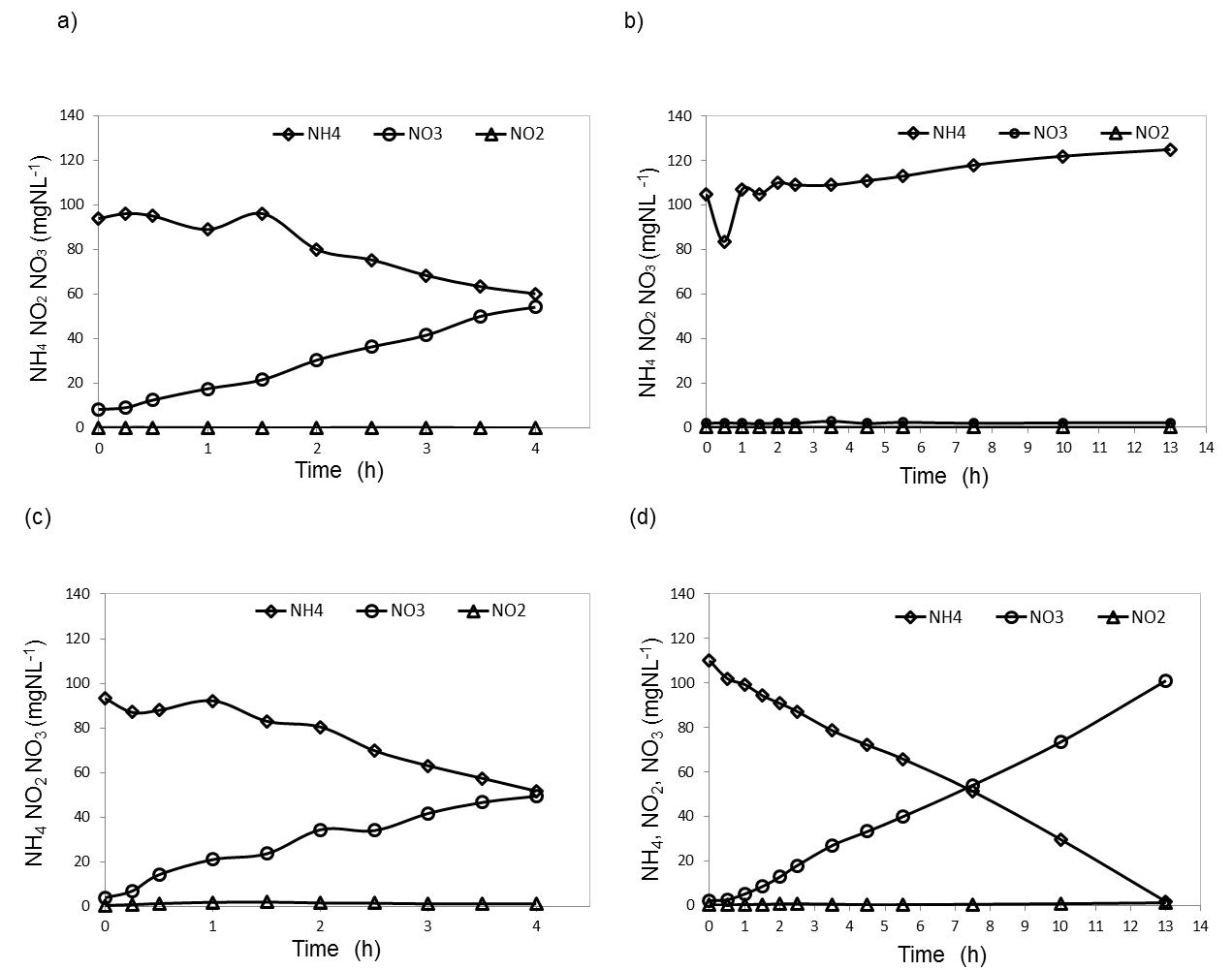


**A) B)**

**C) D)**

**Figure S1**. Capacity test of freshwater culture with freshwater medium (A) and seawater culture with seawater medium (C).Toxicity test of freshwater culture with seawater medium (B) and seawater culture with freshwater medium (D). Note differences on time axis.


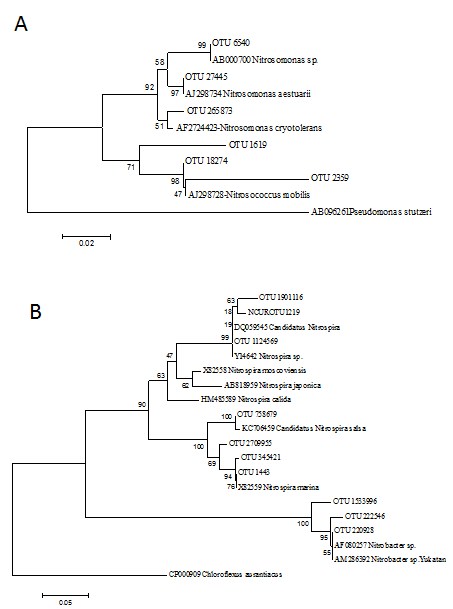


**Figure S2**. A. Phylogenetic relationships of AOB OTUs (A) and NOB OTUs (B). The tree was inferred using the Neighbor-Joining method. The bootstrap consensus tree inferred from 1000 replicates is taken to represent the evolutionary history of the sequences analyzed. The percentage of replicate trees in which the associated sequences clustered together in the bootstrap test (1000 replicates) are shown next to the branches The evolutionary distances were computed using the Maximum Composite Likelihood method and are in the units of the number of base substitutions per site.


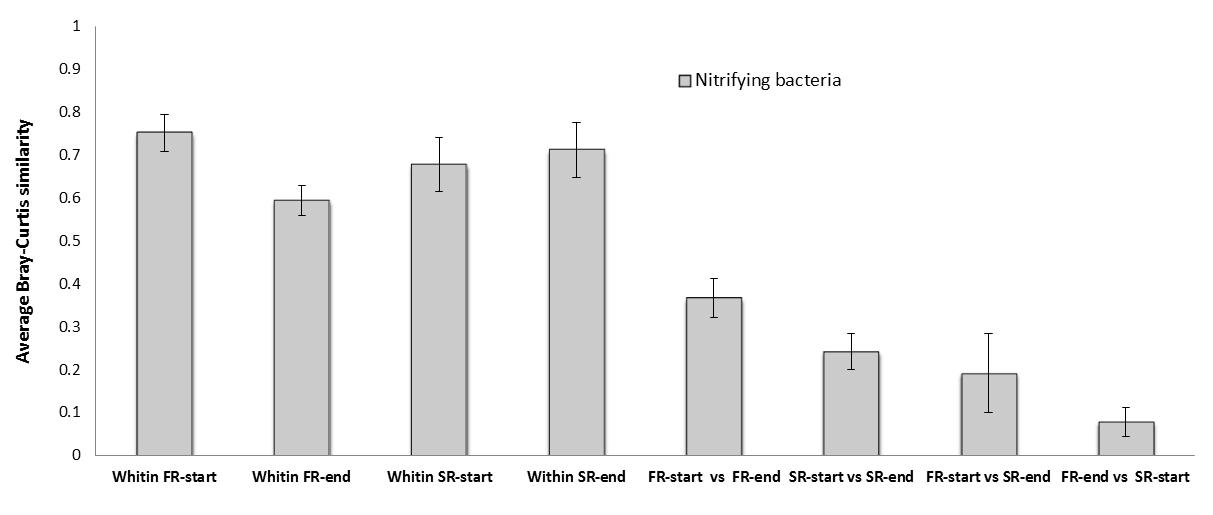


**Figure S3.** Average of Bray-Curtis similarities within and between clusters. The error bars are the standard deviation. One-way ANOSIM indicate significant differences between the groups of samples (p<05).
